# Primary care provider beliefs and knowledge of prescribing gender-affirming hormone therapy to transgender and gender diverse patients

**DOI:** 10.1186/s12875-024-02599-8

**Published:** 2024-10-16

**Authors:** Allison T. Yip, Stacy Charat, Jordan Silva, Jill Blumenthal

**Affiliations:** 1 Department of Medicine, University of California San Diego, 220 Dickinson Street, Suite A, San Diego, CA 92103 USA; 2 Present Address: Department of Medicine, University of California Los Angeles, 200 Medical Plaza, Suite 530, Los Angeles, CA 90024 USA; 3 Primary Care, Veterans Affairs San Diego Health Care, 3350 La Jolla Village Drive, San Diego, CA 92161 USA

**Keywords:** Gender Affirming Hormone Therapy, Transgender, Gender Diverse, Nonbinary, Primary Care, Gender Health, Medical Training

## Abstract

**Background:**

Transgender patients often cannot access a provider who is knowledgeable about providing gender-affirming hormone therapy (GAHT). This study evaluated primary care provider (PCP) comfort and experience with, opinions about, and knowledge of prescribing GAHT to adults.

**Methods:**

An anonymous Qualtrics survey was distributed to PCPs in San Diego County. Fisher’s exact test assessed any association between age, years in practice, or practice setting and 1) comfort in prescribing GAHT and 2) favorable statements about learning about, providing, and benefitting from training in GAHT. T-tests determined relationship between age, years in practice, or practice type setting with number of correctly answered multiple choice knowledge-based questions out of 4.

**Results:**

Out of 220 responses, median age was 41, 60% had practiced for ≤ 10 years, and 19% had practiced in an academic setting. Forty-two percent did not receive any education about transgender healthcare during medical training. The most commonly reported barrier to providing GAHT was lack of training (74%). PCPs age ≤ 41 (67% vs 49%, *p* = 0.009), PCPs practicing for ≤ 10 years (65% vs 51%, *p* = 0.037), and PCPs in non-academic settings (64% vs. 41%, *p* = 0.013) were more likely to report being comfortable with prescribing GAHT. PCPs age ≤ 41 (89% vs 62%, *p* < 0.001) and PCPs practicing for ≤ 10 years (86% vs 66%, *p* < 0.001) were more likely to show interest in learning about GAHT. PCPs age ≤ 41 (74% vs 46%, *p* < 0.001) and PCPs practicing for ≤ 10 years (70% vs 50%, *p* = 0.003) were more likely to show interest in prescribing GAHT. Knowledge scores were higher for PCPs age  ≥42 (mean 1.7 vs 1.4, *p* = 0.033) and PCPs working in academic centers (mean 2.0 vs 1.4, *p* = 0.002).

**Conclusion:**

Younger (age ≤ 41) and early career (practicing for ≤ 10 years) PCPs reported being more comfortable with prescribing GAHT and had more favorable opinions in learning about, providing, and benefitting from training in GAHT. They are interested in providing GAHT; however, few prescribe GAHT with most reporting lack of training as a major barrier. This was evident with overall low knowledge scores regardless of age, experience, or clinical setting and underscores the need for increased educational efforts in transgender care throughout medical training.

**Supplementary Information:**

The online version contains supplementary material available at 10.1186/s12875-024-02599-8.

## Background

Transgender and gender diverse (TGD) individuals have a gender identity and/or gender expression that differs from social and cultural norms of what is expected for that sex assigned at birth [1]. TGD patients experience disparities in health outcomes and are an underserved population in healthcare [2, 3]. Many TGD individuals delay seeking healthcare due to concerns about stigma and discrimination [4]. Transgender stigma limits access to healthcare resources and operates at the individual, interpersonal, and structural levels to impact health; examples of structural stigma include societal norms, stigmatizing policies, lack of provider training and education, healthcare access barriers, as well as economic and gender inequality [5]. Sociopolitical factors influence much of the United States, and for many, there are no laws to protect against discrimination of the TGD community or to protect access to TGD healthcare. Early insights from the 2022 U.S. Transgender Survey report that of those who saw a healthcare provider within the last year, nearly one-half of respondents aged 18 or older reported having at least one negative experience because they were transgender. Furthermore, 24% of respondents did not see a doctor within the last 12 months due to fear of mistreatment [6].

TGD individuals experience many barriers to accessing routine and evidence-based healthcare. Gender-affirming care, as defined by the World Health Organization, includes a range of social, psychological, behavioral, and medical interventions, including hormonal treatment or surgery, aimed at supporting and affirming an individual’s gender identity [7]. One of the most commonly reported barriers is the limited number of providers who are sufficiently knowledgeable about the healthcare needs of this population and provide gender-affirming care [8–11], which may, in part, stem from a deficiency in medical education about transgender care across all stages of medical training [11]. In one survey of internal medicine residents at a large urban academic center, less than half reported any education in transgender health; however, 97% of these same residents agreed it was valuable for an internist to learn more about the care of transgender individuals [12]. According to the 2018 Council of Academic Family Medicine's Educational Research Alliance survey of United States and Canadian Family Medicine clerkship directors, 72% of family medicine clerkship directors agreed that transgender healthcare should be a required part of the medical school curriculum; however, only 26% agreed they were comfortable teaching transgender healthcare to medical students [13].

An important aspect of healthcare for many TGD patients is gender-affirming hormone therapy (GAHT), which provides feminizing or masculinizing physical changes that are more congruent with a patient’s gender identity. With appropriate training, GAHT can be managed by a variety of providers, including advanced practice providers and primary care providers (PCPs) [1, 14, 15]. Receiving GAHT improves health outcomes for TGD patients. For example, GAHT has positive psychological effects, such as reducing symptoms of anxiety and depression, lowering perceived and social distress, and improving quality of life and self-esteem [16, 17]. Moreover, studies have shown that TGD patients living with HIV are more likely to be virally suppressed and retained in care when receiving GAHT [18]. Early insights from the 2022 U.S. Transgender Survey reported that 84% of respondents currently receiving hormone treatment were “a lot more satisfied” with their life [6]. Due to the positive impact of GAHT on TGD individuals, PCPs across practices should be well-informed about and able to offer GAHT to their patients. The WPATH Standards of Care Version 8 affirms that GAHT falls within the scope of primary care and that with appropriate training, GAHT can be managed by most PCPs [1].

There have been published reports surveying medical providers and their knowledge of caring for the TGD population. In a study interviewing eleven nurse practitioners, a main theme was that their personal and professional knowledge gaps threatened their ability to deliver quality, patient-centered care to TGD patients despite their best intentions [19]. In one national cross-sectional survey of obstetrics and gynecology (OBGYN) providers, one-third of providers were not knowledgeable about the steps that TGD patients must take to undergo gender-affirming surgeries, and less than half of providers were familiar with recommendations for routine health maintenance for these patients [20]. Medical providers and their attitudes and protocols for caring for the TGD population have varied. In a survey of OBGYN providers in a single healthcare system, only 27.6% of respondents were willing to initiate GAHT for transgender patients, and this willingness was associated with younger age and being a resident physician [21]. Another survey study of endocrinologists assessed the protocols of those who prescribe GAHT and found that 42.9% of respondents reported that their practice required documentation of psychosocial evaluation from a mental health professional before initiating GAHT [22]. The WPATH Standards of Care Version 8 recommended that health care professionals should not make it mandatory for TGD individuals to undergo psychotherapy prior to the initiation of gender-affirming treatments while acknowledging that psychotherapy may be helpful for some TGD patients [1]. Notably, there are few published reports specifically surveying PCPs. In one survey of PCPs in a midwestern health system, 85.7% were willing to provide routine care to TGD patients, and the willingness to provide routine care decreased with age [23]. At this same midwestern health system, another online survey of internal and family medicine physicians and residents revealed only approximately half of PCPs were willing to continue GAHT for TGD patients [24]. This study specifically targeted PCPs within San Diego County to assess their comfort and experience with, as well as their knowledge of prescribing GAHT to adult TGD patients; to our knowledge, this is the only survey of this kind within this neighborhood.

## Methods

The survey was created on Qualtrics. The survey began with two qualifying questions to ensure that the respondents were practitioners who met the following inclusion criteria: 1) had completed training and were currently in practice and 2) provided primary care health services to adult patients. The survey additionally collected data on demographics (5 items), clinical practice setting (2 items), transgender healthcare training and hormone-prescribing practices (5 items), comfort in prescribing GAHT (1 item), barriers to prescribing GAHT (1 item), and respondent’s perception of their own knowledge regarding the healthcare needs of TGD patients (1 item). A 6-point Likert scale assessed agreement with statements regarding personal interest in formal transgender healthcare training (4 items). The full survey is attached to the Appendix. Knowledge scores were calculated from the total number of correctly answered multiple choice questions out of four evaluating knowledge of GAHT treatment guidelines for transgender patients (0 = lowest score, 4 = highest score). These four knowledge questions were created by the investigators as there is currently no validated method to assess knowledge of GAHT.

The anonymous online Qualtrics survey was sent to representatives at 30 healthcare organizations in San Diego County. A diverse range of institutions were contacted, including small private practices, large multispecialty groups, academic medical centers, healthcare startups, community health centers, and a local professional PCP organization. This list of 30 unique practices was created after aiming to contact all major academic and private PCP groups plus smaller group PCP practices in San Diego County. The Kaiser Permanente medical group deferred participation in this study because their policies required independent IRB approval. We were unable to find a comprehensive list of solo-practice PCPs, but the survey link was included in the San Diego County Medical Society newsletter. Smaller primary group practices within San Diego County were identified using the Google Search engine and selected if there was an associated website with contact information. Of the 30 institutions contacted, 19 agreed to participate and forwarded the anonymous survey link to their PCP groups via email for optional completion. The Qualtrics survey link allowed only one response per IP address as a way to avoid duplicate responses.

Data was collected from December 8th, 2021 to June 30th, 2022. Initially only the first 50 survey respondents were offered a $10 electronic Amazon gift card (from 12/8/21–01/17/22), but in an effort to increase survey responses, the IRB was revised and approved for all participants after 02/23/22 to receive a gift card for participation. Survey participants who claimed a giftcard for completion of the survey were redirected to a separate Qualtrics survey to provide an email in order to decouple responses to any identifiable data. These email addresses were not accessible to those performing the statistical analysis to ensure that the survey responses were anonymous.

Descriptive statistics characterized demographic data and the number of correctly answered knowledge-based questions. Respondents were asked to rank up to three barriers to prescribing GAHT; we report the percentage of participants who included each item within their top three barriers. Fisher’s exact test was used to assess the association between age, years in practice, or practice setting with reported comfort in prescribing GAHT as well as favorable statements about learning about, providing, and benefitting from graduate medical education (GME) or continuing medical education (CME) in GAHT. Fisher’s exact test was chosen for analysis of categorical data because of the small sample size to represent PCPs in San Diego County. A dichotomous outcome was created where responses of ‘strongly disagree,’ ‘disagree,’ and ‘neutral,’ were considered unfavorable and all other responses were considered favorable. Further explanation of why a dichotomous outcome was chosen is included in the Appendix. T-tests were used to evaluate any relationship between age, years in practice, or practice setting and knowledge scores out of 4.

The Institutional Review Board at the University of California, San Diego approved this study. Prior to starting the survey, all participants viewed and accepted an online statement for obtaining informed consent.

## Results

### Study participants

Two-hundred and twenty unique and complete survey responses were included in the analysis. There were 252 total responses; 230 met inclusion criteria and 10 responses were not included as they were only partially complete. Survey respondents included doctors of allopathic medicine (MDs), doctors of osteopathic medicine (DOs), and advanced practice providers including physician assistants (PAs) and nurse practitioners (NPs). Median age was 41 (IQR 11); thus, younger PCPs were defined as PCPs age ≤ 41, whereas older PCPs were defined as age  ≥42. Sixty-four percent identified as women, 64% as white, and 86% as non-Hispanic; 62% percent held an MD/DO degree. Twenty percent practiced at a multispecialty medical group practice, 19% practiced at an academic university health system. Sixty percent reported being in practice for 10 years or less; in this manuscript, early career PCPs are defined as PCPs in practice ≤ 10 years vs late career PCPs are defined as PCPs in practice > 10 years. Seventy-four percent of PCPs reported taking care of ≤ 10 transgender patients within the last year (see Table 1). Forty-two percent (42%) reported not receiving any education about transgender healthcare during medical school, residency, or fellowship training, and 60% received additional education about transgender healthcare after completing formal training. Sixty-seven percent (67%) worked in a clinic that offered GAHT, but only 34% prescribed GAHT to their patients.

**Table 1:** Participant demographics and healthcare experience

Descriptive Data, n (%)			
**Healthcare role** (*n* = 220)		**Practice care setting** (*n* = 220)	
MD/DO	137 (62%)	Academic university health system	41 (19%)
NP/PA	80 (36%)	Federally qualified health center	34 (15%)
Other	3 (2%)	Individual private practice	38 (17%)
		Multispecialty medical group practice	45 (20%)
**Gender Identity** (*n* = 220)		Veteran’s Health Administration	15 (7%)
Woman	140 (64%)	Managed Care organization	8 (4%)
Man	73 (33%)	Other	39 (18%)
Transgender/Nonbinary	1 (< 1%)		
Prefer not to answer	6 (3%)		
**Race** (*n* = 220)		**Years in Practice** (*n* = 220)	
White	140 (64%)	0–5 years	65 (30%)
Black or African America	15 (7%)	6–10 years	65 (30%)
Asian	43 (20%)	11–15 years	38 (17%)
Mixed	14 (6%)	16–20 years	12 (5%)
Prefer not to answer	7 (3%)	> 20 years	40 (18%)
Native Hawaiian or other Pacific Islander	1 (< 1%)		
Not sure	0 (0%)		
**Ethnicity** (*n* = 220)		**# of TGD patients cared for in the last year** (*n* = 220)	
Non-hispanic	190 (86%)	0	17 (8%)
Hispanic or Latinx	24 (11%)	1–10	162 (74%)
Not sure	2 (< 1%)	11–50	39 (18%)
Prefer not to answer	4 (2%)	> 50	2 (< 1%)

### Barriers to providing GAHT

**Fig. 1:**
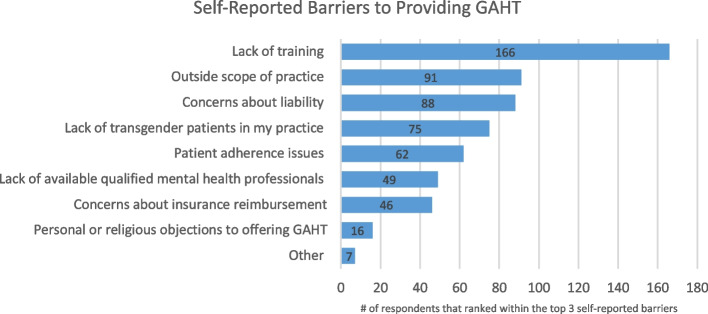
Self-Reported Barriers to Providing GAHT Legend: The survey asked respondents to rank up to three choices as the top three barriers to providing gender-affirming hormone therapy (GAHT). Data is displayed in composite in the bar graph

The most commonly reported barriers in providing GAHT were lack of training (75%), outside scope of practice (41%), concerns about liability (40%), lack of TGD patients in their practice (34%), and patient adherence issues (28%) (see Fig. 1).

### Comfort and interest in prescribing GAHT

With regard to question 15 in the survey (see Appendix A), younger PCPs age ≤ 41 vs older PCPs age  ≥42 years (67% vs 49%, *p* = 0.009), early career PCPs who have been in practice ≤ 10 years vs > 10 years (65% vs 51%, *p* = 0.037), and PCPs working in non-academic settings vs academic settings (64% vs. 41%, *p* = 0.013) were more likely to report being comfortable with prescribing GAHT. Detailed information on responses to question 15 are provided in a separate figure in Appendix C. Similarly with regard to questions 17a-b (see Appendix A), younger PCPs compared to older PCPs (89% vs 62%, *p* < 0.001) and early career PCPs compared to late career PCPs (86% vs 66%, *p* < 0.001) were more likely to show interest in learning about GAHT. Moreover, younger PCPs compared with older PCPs (74% vs 46%, *p* < 0.001) and early career PCPs compared to their seniors (70% vs 50%, *p* = 0.003) were more likely to show interest in prescribing GAHT. There was no association between practice setting and opinion regarding learning about or prescribing GAHT (see Table 2).

### Training and knowledge in prescribing GAHT

With regard to survey questions 17c-d (see Appendix A), most PCPs answered favorably regarding the benefit of having formal GME and CME in GAHT, 86% and 85%, respectively. There was no association between age, years in practice, or practice setting and opinion regarding the benefit of having GME or CME in GAHT. Knowledge scores were higher for older PCPs compared to younger PCPs (mean 1.7 vs 1.4, *p* = 0.033) and academic compared to non-academic providers (mean 2.0 vs 1.4, *p* = 0.002). There was no significant difference between years in practice and knowledge score (Table 2). Of the 220 respondents, 16% answered zero knowledge questions correctly, 35% answered one knowledge question correctly, 28% answered two knowledge questions correctly, 17% answered three knowledge questions correctly, and 4% answered all four knowledge questions correctly.

**Table 2:** Predictors of comfort, interest, training and knowledge of prescribing GAHT

	**PCP age:**			**#Years in practice**			**Practice setting**		
**Group**	**age** $\le$ **41**	**age ≥42**	***p*-value†**	**0–10 years**	** > 10 years**	***p*-value†**	**Non-academic**	**Academic**	***p*-value†**
**% comfortable prescribing GAHT (n)**	67% (123)	49% (97)	0.009*	65% (130)	51% (90)	0.037*	64% (179)	41% (41)	0.013*
**% answered positively re: learning more about GAHT (n)**	89% (123)	63% (97)	< 0.001*	86% (130)	66% (90)	< 0.001*	76% (179)	78% (41)	0.683
**% answered positively re: interest in prescribing GAHT (n)**	74% (123)	46% (97)	< 0.001*	70% (130)	50% (90)	0.003*	63% (179)	59% (41)	0.722
**% answered positively re: the benefit of GME on GAHT (n)**	89% (123)	82% (97)	0.242	87% (130)	84% (90)	0.694	85% (179)	86% (41)	1.00
**% answered positively re: benefit of CME on GAHT (n)**	87% (123)	81% (97)	0.267	85% (130)	85% (90)	1.00	83% (179)	85% (41)	0.811
			***p*-value‡**			***p*-value‡**			***p*-value‡**
**Correctly answered knowledge questions (n)**	1.4 (123)	1.7 (97)	0.033*	1.4 (130)	1.7 (90)	0.09	1.4 (179)	2 (41)	0.002*

^*^statistically significant *p*-value

^†^Fisher’s Exact test *p*-value

^‡^T-test *p*-value

## Discussion

This study is the first of its kind in California to survey PCPs on their experience in, training in, and practice of GAHT. In 2014, there were an estimated 1.4 million adults in the United States who identified as transgender, with 218,400 transgender adults residing in California, comprising the largest number of transgender adults as compared to any other state [25]. San Diego is the second largest county within California, and it is important to understand the gender health practices of its PCPs.

This study suggests there are few PCPs in San Diego that are comfortable prescribing GAHT. Although a majority of survey participants had taken care of at least one transgender patient in their practice within the last year, only two-thirds of the respondents worked in a clinic that provided GAHT, and even fewer prescribed GAHT. This finding revealed that only a small percentage of the surveyed PCPs in San Diego actually prescribe GAHT and that TGD patients in San Diego would likely need a separate provider who could prescribe GAHT. Patients who express desire for GAHT may not be able to easily find prescribing providers within primary care and may need referrals to specialists or gender health clinics.

Having to go to another provider for GAHT can be a barrier for TGD patients and could disincentivize them from seeking any medical care at all. Ideally, a single provider could address primary care needs as well as transgender healthcare for a TGD individual. For example, a pilot clinic providing GAHT within primary care in New Zealand interviewed patients and providers and confirmed the value of this practice model as it increased accessibility, depathologized gender diversity, and reduced wait times [26]. Our data reaffirms there are not enough PCPs who feel comfortable prescribing GAHT. PCPs are often the first point of contact for patients naïve to the healthcare setting and for TGD patients, which may be the only time they interact with a medical system. In a community-based participatory study with fifty TGD individuals in New York City, participants consistently reported that medical providers’ inadequate knowledge of transgender health issues and lack of cultural competency were significant and persistent barriers to healthcare utilization, even for routine preventative services [27]. Undoubtedly, it would be beneficial to have PCPs with the cultural competence to appropriately care for the TGD community; however, our data revealed that only 43% of survey participants felt at least somewhat knowledgeable about the unique healthcare needs of this population.

Moreover, the PCPs in San Diego within this survey found it difficult to provide GAHT due to inexperience and lack of training. Although more than half of respondents reported having some education about transgender healthcare during their medical training with additional post-graduate training, this training may not be sufficient to prepare PCPs to be proficient in prescribing GAHT. These results are similar to another large online study of healthcare providers across four different European countries, which showed that more than half reported some form of training on transgender healthcare, with training having a significant effect on confidence level for health care providers. Furthermore, this study revealed that nearly all providers believed that training would increase their competence [28]. Interestingly, the knowledge scores for every subgroup within our study were relatively low regardless of age, experience, or clinical setting, indicating that all individuals could benefit from more formal education to increase competency in prescribing GAHT.

Younger, early career PCPs reported being more comfortable prescribing GAHT and had more favorable opinions in learning about, providing, and benefitting from further training in GAHT for TGD individuals. This finding suggests that more recent generations of healthcare providers recognize the importance of providing GAHT, as TGD issues have become more prominent within current events and mainstream culture. Surprisingly, despite younger PCPs reporting feeling more comfortable with TGD healthcare, it was actually PCPs in academic settings and older PCPs who scored higher on knowledge-based survey questions. However, these were small sample sizes and may not be clinically relevant findings. As discussed previously, the overall low knowledge scores highlight the need to focus on improving medical training. A 2018 published literature review on medical education and transgender health yielded 119 papers with an additional 12 citations which showed that transgender health has yet to gain widespread curricular exposure [29]. Currently, there are nascent efforts toward incorporating it in both undergraduate and graduate medication education with curriculum largely composed of one-time attitudes and awareness-based interventions, which show significant short-term improvements but fail to emphasize clinical skills, evaluate patient outcomes, and lack longer term assessments [29]. The consensus in existing literature support addressing this training gap more formally, and it is recommended that transgender medical education shift toward teaching interventions that are longitudinally integrated and clinical skills based [29]. The WPATH Standards of Care Version 8 recommends providers become knowledgeable and get training where possible about the healthcare needs of TGD people, including the benefits and risks of gender-affirming care as a core principle in delivering competent services to this population [1]. A list of recommended resources for PCPs is included in the Appendix.

There are several limitations to this study. A survey-based study with voluntary participation inherently elicits selection bias, capturing those who have strong opinions or personal experience in this topic, which may not represent current prescribing practices of all PCPs. There was also limited number and diversity of responses, making the generalizability of results difficult. The survey in this study was not piloted or validated with PCPs prior to distribution; additionally, the knowledge questions have not been validated as there is currently no standardized method to assess knowledge regarding GAHT. The survey in this study was based on a prior national survey study on GAHT distributed to practitioners who care for patients living with HIV, and the knowledge based questions were reviewed by experts in the field prior to distribution [30]. Because this survey was distributed anonymously through listservs and representatives from large PCP groups, there was no way to calculate a response rate. Larger studies will be important to obtain more accurate reflections of current practice and attitudes regarding GAHT within PCPs.

## Conclusions

Younger, early career PCPs reported being more comfortable with prescribing GAHT and had more favorable opinions regarding learning about, providing, and benefitting from training in GAHT for TGD individuals. Although PCPs in non-academic settings reported feeling comfortable prescribing GAHT, PCPs in academic settings and older PCPs scored higher on knowledge-based survey questions. There is promising interest among younger, early career PCPs in providing GAHT to TGD patients; however, a minority actually prescribe GAHT with most reporting lack of training as a major barrier. This is evident with overall low knowledge scores regardless of age, experience, or clinical setting. Furthermore, most PCPs agreed they would benefit from continuing education on GAHT. These results underscore the need for increased educational efforts in transgender care throughout medical training for primary care providers, including CME.

## Supplementary Information

Supplementary Material 1.

Supplementary Material 2.

Supplementary Material 3.

Supplementary Material 4.

Supplementary Material 5.

## Data Availability

All data collected during the current study are available from the corresponding author upon reasonable request.

## Abbreviations

GAHT: Gender affirming hormone therapy
PCP: Primary care provider
TGD: Transgender and gender diverse
WPATH: World Professional Association for Transgender Health
GME: Graduate medical education
CME: Continuing medical education
