# Supplementary material for: Primary care provider beliefs and knowledge of prescribing gender-affirming hormone therapy to transgender and gender diverse patients
Source: BMC Prim Care. 2024 Oct 16;25:372. doi: 10.1186/s12875-024-02599-8 (PMC11481314; doi:10.1186/s12875-024-02599-8)
Supplement: Supplementary file 1 — Supplementary Material 1. [file 12875_2024_2599_MOESM1_ESM.docx]

**Appendix A**

**Transgender Health Survey for Primary Care Providers**

We are interested in evaluating how primary care practitioners offer gender-affirming hormone treatment and how comfortable they are with this process in order to assess potential gaps in transgender healthcare. More specifically, we aim to identify gaps in transgender healthcare knowledge and to advocate for improvements in LGBTQ medical education. If we understand where gaps arise for those practicing, we can better target the areas of improvement for trainees. For the purpose of this survey, we consider transgender individuals to be anyone who self-identifies as transgender or nonbinary.

1. Do you provide primary care health services to the patients you care for?
   1. Yes
   2. No
2. What is your primary healthcare role?
   - 1. MD/DO
     2. NP/PA
     3. Other
3. What is your age? _ _
4. What is your gender identity?
   - 1. Female
     2. Male
     3. Transfemale
     4. Transmale
     5. Nonbinary
     6. Prefer not to answer
5. What is your race?
   - 1. White
     2. Black or African American
     3. Asian
     4. American Indian or Alaska Native
     5. Native Hawaiian or other Pacific Islander
     6. Mixed
     7. Not sure
     8. Prefer not to answer
6. What is your ethnicity?
   - - 1. Non-Hispanic
       2. Hispanic or Latino/a
       3. Not sure
       4. Prefer not to answer
7. What is your practice care setting?
   1. Academic university health system
   2. Federally qualified health center
   3. Individual private practice
   4. Multispecialtiy medical group practice
   5. Veteran’s Health Administration
   6. Managed Care Organization
   7. Other
8. How many years have you been in practice after completing your highest level of training (residency or fellowship)?
   1. 0-5
   2. 6-10
   3. 11-15
   4. 16-20
   5. >20
9. During medical school, residency, or fellowship training, did you receive training about transgender healthcare?
   1. No
   2. Yes, I received general training about health issues impacting transgender patients.
   3. Yes, I received detailed training about health issues impacting transgender patients, *including education on prescribing gender-affirming hormone therapy*.
   4. Not sure
10. Since you completed your formal training, have you received additional education about transgender healthcare?
    1. No
    2. Yes, I have since received general training about health issues impacting transgender patients.
    3. Yes, I have since received detailed training about health issues impacting transgender patients, *including education on prescribing gender-affirming hormone therapy.*
    4. Not sure
11. In the last year, how many transgender patients have you cared for in the outpatient setting?
    1. None
    2. 1-10
    3. 11-50
    4. 51-100
    5. >100
12. Does your clinic offer gender-affirming hormone therapy to transgender patients?
    - 1. No
      2. Yes
13. If ‘Yes’ to the prior question, do you offer gender-affirming hormone therapy to these patients?
    - - 1. No
        2. Yes
14. How comfortable are you prescribing gender-affirming hormone therapy to transgender patients?
    1. Very comfortable, I prescribe them myself.
    2. Comfortable, but usually ask for help from an expert.
    3. Comfortable, but I have not yet prescribed.
    4. Not comfortable, I do not prescribe hormone therapy.
15. What, if any, barriers do you face in providing gender-affirming hormone therapy to transgender patients? *Please rank up to three choices numerically (1, 2, 3) for your top three barriers.*
16. Lack of training
17. Outside scope of practice
18. Patient adherence issues
19. Concerns about liability
20. Concerns about insurance reimbursement
21. Lack of available qualified mental health professionals
22. Lack of transgender patients in my practice
23. Personal or religious objections to offering gender-affirming hormone therapy
24. Other:_________
25. Please rate your agreement with the following statements:
26. I am interested in learning more about gender-affirming hormone therapy for transgender patients.
    - 1. Strongly agree
      2. Somewhat agree
      3. Neutral
      4. Somewhat disagree
      5. Strongly disagree
      6. I am already knowledgeable about gender-affirming hormone therapy.
27. I am interested in prescribing gender-affirming hormone therapy to my transgender patients.
28. Strongly agree
29. Somewhat agree
30. Neutral
31. Somewhat disagree
32. Strongly disagree
33. I already prescribe gender-affirming hormones to my patients
34. It would be beneficial to have formal training in gender-affirming hormone therapy practices during residency or fellowship.
35. Strongly agree
36. Somewhat agree
37. Neutral
38. Somewhat disagree
39. Strongly disagree
40. I received formal training in hormone therapy practices during residency or fellowship.
41. It would be beneficial to have continuing medical education in gender-affirming hormone therapy practices after completion of residency or fellowship.
42. Strongly agree
43. Somewhat agree
44. Neutral
45. Somewhat disagree
46. Strongly disagree
47. I currently receive or have access to continuing medical education in gender-affirming hormone therapy practices.
48. How knowledgeable do you feel about the overall unique healthcare needs (e.g., gender-affirming hormone therapy, disease or cancer screening, gender-affirming surgeries) for transgender patients?
49. Very knowledgeable
50. Somewhat knowledgeable
51. Neutral
52. Not particularly knowledgeable
53. Not at all knowledgeable

We would like to ask you some questions to gauge your knowledge of gender-affirming hormone therapy.

Top of Form

1. Most transgender women experience a maximal effect of feminizing hormone therapy within:
2. 2-3 days of starting therapy
3. 2-3 weeks of starting therapy
4. 2-3 months of starting therapy
5. 2-3 years of starting therapy
6. What possible side effect of testosterone is closely monitored during masculinizing hormone therapy?
7. Stroke
8. Prolactinoma
9. Polycythemia
10. Osteopenia/osteoporosis
11. Which route is **not commonly** used to give estrogen as feminizing hormone therapy?
12. Oral
13. Intramuscular
14. Subcutaneous
15. Transdermal
16. A transwoman comes to see you in clinic and is found to have an elevated prolactin level? Which of the following medications is likely to have caused this laboratory abnormality?
17. Finasteride
18. Estradiol
19. Spironolactone
20. Leuprolide
