# Supplementary material for: Primary care provider beliefs and knowledge of prescribing gender-affirming hormone therapy to transgender and gender diverse patients
Source: BMC Prim Care. 2024 Oct 16;25:372. doi: 10.1186/s12875-024-02599-8 (PMC11481314; doi:10.1186/s12875-024-02599-8)
Supplement: Supplementary file 3 — Supplementary Material 3. [file 12875_2024_2599_MOESM3_ESM.docx]

**Appendix C) Dichotomous Outcomes**

A dichotomous outcome was created where ‘strongly disagree’, ‘disagree’ and ‘neutral’ responses were considered unfavorable and all other responses were considered favorable. This was decided post-hoc and was done instead of comparing mean Likert-scale scores. The reason why this method was chosen is best described with an example using one of our Likert-scale questions.

For example, in the question below:

I am interested in learning more about gender-affirming hormone therapy for transgender patients.

1. Strongly agree
2. Somewhat agree
3. Neutral
4. Somewhat disagree
5. Strongly disagree
6. I am already knowledgeable about gender-affirming hormone therapy.

There are six answer choices with the last choice stating “I am already knowledgeable about gender-affirming care”. We thought it was important to create this as an answer choice because it distinguishes between agreement with the statement versus existing expertise. However, when considering statistical analysis, choosing the sixth answer choice may not fit appropriately into the rank order to be scored as 6 points when compared to “strongly agree” which would be 5 points. Because of the nuance between the choices, it was decided that it would be more appropriate to dichotomize the data as unfavorable vs favorable.”
