# Supplementary material for: Primary care provider beliefs and knowledge of prescribing gender-affirming hormone therapy to transgender and gender diverse patients
Source: BMC Prim Care. 2024 Oct 16;25:372. doi: 10.1186/s12875-024-02599-8 (PMC11481314; doi:10.1186/s12875-024-02599-8)
Supplement: Supplementary file 4 — Supplementary Material 4. [file 12875_2024_2599_MOESM4_ESM.docx]

**Appendix D) Comfort in Prescribing GAHT**

Detailed responses to survey question 15
