# Supplementary material for: Primary care provider beliefs and knowledge of prescribing gender-affirming hormone therapy to transgender and gender diverse patients
Source: BMC Prim Care. 2024 Oct 16;25:372. doi: 10.1186/s12875-024-02599-8 (PMC11481314; doi:10.1186/s12875-024-02599-8)
Supplement: Supplementary file 5 — Supplementary Material 5. [file 12875_2024_2599_MOESM5_ESM.docx]

**Appendix E) Recommended Resources**

Resources for those interested in learning more about gender-affirming care:

- WPATH World Professional Association for Transgender Health Standards of Care Version 8
- UCSF Guidelines for the Primary and Gender-Affirming Care of Transgender and Gender Nonbinary People
- Endocrinology Society Clinical Practice 2017 guidelines on endocrine treatment of gender incongruent persons
- Fenway Institute National LGBTQIA+ Health Education Center
- GLMA Health Professionals Advancing LGBTQ+ Equality
